# Prospective evaluation of hematological parameters in preoperative renal cell cancer patients

**DOI:** 10.1186/s12894-022-01118-0

**Published:** 2022-12-10

**Authors:** Ozden Demir, Guzin Demirag, Gokhan Aslan

**Affiliations:** 1 Faculty of Medicine, Department of Medical Oncology, Ondokuz Mayıs University, Samsun, Turkey; 2 Faculty of Medicine, Department of Internal Medicine, Ondokuz Mayıs University, Samsun, Turkey

**Keywords:** Renal cell cancer, Fibrinogen to albumin ratio, Biomarkers, Prognostic factor

## Abstract

**Background:**

Of all the genitourinary cancers, renal cell carcinoma (RCC) is still the most common malignancy with high mortality rates. There are still insufficient biomarkers to predict disease prognosis. Systemic inflammation markers play an important role in tumor development and growth. There are studies which show the relationship of fibrinogen and albumin individually with cancer prognosis in many cancers. Fibrinogen/albumin ratio(FAR), on the other hand, has prognostic importance like other inflammation indicators in cancer. Therefore, we investigated whether FAR had a potential value in evaluating the prognosis of patients with nonmetastatic kidney cancer or not.

**Methods:**

A total of 72 patients who had nephrectomy operation at 19 Mayıs University, Faculty of Medicine between January 2019 and January 2021 and who did not have distant metastasis were included in the study. FAR was calculated from the blood taken from the patients before the nephrectomy operation. The cut-off value was found for this FAR by receiver operating characteristic(ROC) curve analysis. The patients were divided into 2 groups as high- and low-FAR according to this cut-off value. Kaplan Meier test was used to evaluate the predictive value of clinicopathological parameters for overall survival (OS). The Log-rank test was used to determine whether there was a relationship between the preoperative FAR and the clinico-pathological data of the patients.

**Results:**

The best cutoff value for the FAR was 0.114. A FAR > 0.114 was associated with higher Fuhrman Grade (FG) (*P* < 0.0001) and later pathological T stage (*P* < 0.0001). Patients with a high FAR (> 0.114) had worse OS [Std. Error 2.932, 95% confidence interval (CI): 73.659–85.154, *P* < 0.0001]. In addition, a positive significant correlation was found between high grade and platelet lymphocyte ratio (*p* < 0,020). Furthermore, a significant correlation was found between the pathology t stage of the patients and the platelet lymphocyte ratio (*p*: 0.020).

**Conclusions:**

The preoperative FAR is an independent prognostic factor of OS in renal cancer patients. A FAR > 0.114 was significantly related to decreased survival in renal cancer patients. In addition, the platelet-lymphocyte ratio seems to be related to OS, as well as FAR. Further studies are required on this subject.

## Background

RCC is one of the most common malignant tumors of the urinary system. It is the 6th most common cancer in men and the 10th in women [1]. RCC has the highest mortality rate of the genitourinary cancers and the incidence of RCC has risen steadily [2]. The reason for the increase in the incidence of RCC is the increased frequency of abdominal imaging in high-income populations and their incidental detection in these images [3]. Approximately one third of the patients diagnosed with RCC are found to be in the advanced stage [4]. In patients who are diagnosed with RCC at an early stage and operated on, the probability of metastasis during follow-up is 30 percent [5]. In RCC, the clinical parameters of the patient are as important as other parameters such as the stage, grade or histological type in determining the prognosis of the disease. A better management of RCC requires identification of molecular changes which affect tumor behavior and clinical outcomes [6].

Inflammation plays a role in every stage of tumor development and increased markers of systemic inflammation have been shown to be associated with survival in various cancers [7–9]. Circulating leukocytes and acute phase proteins are often used as markers of systemic inflammation. Neutrophil–lymphocyte ratio (NLR) is the most commonly used leukocyte-based inflammation marker. NLR has been shown to have prognostic significance in many cancers, including colorectal cancer. In addition, it has been shown that lymphocyte-monocyte ratio (LMR), platelet-lymphocyte ratio (PLR), systemic-immune-inflammation index (SII) are significantly associated with prognosis in various cancers [10–14].

Fibrinogen and albumin are frequently measured tests in clinical practice. Fibrinogen is both an indicator in the coagulation cascade and an acute phase protein that increases in systemic inflammation [15]. Albumin is a negative acute phase protein that decreases in systemic inflammation [16]. Furthermore, elevated plasma fibrinogen levels and increased FAR have been shown to be associated with reduced survival in patients with cancer [15–18]. In addition, Grahim et al. showed that fibrinogen has as much prognostic significance as CRP in malignant pleural mesothelioma [15]. Information on the prognostic significance of fibrinogen and albumin individually or in combination in cancer is still not sufficient[19, 20]. Our hypothesis is that FAR may have a potential value in predicting the prognosis of patients with RCC. In addition, using FAR to evaluate prognosis can be inexpensive, usable, and easily reproducible.

This study aimed to evaluate the prognostic value of a preoperative FAR in patients RCC and to compare it with established systemic inflammation markers, including NLR, LMR, PLR, hemoglobin-RDW(Red Cell Distribution Width) ratio (HRR) and SII. We found that there were few studies on the subject in the literature.

## Methods and material

A total of 72 patients with renal cancer who underwent radical nephrectomy between 2019 and 2021 were collected from the Urology Department of 19 May University Faculty of Medicine Hospital. All cases were confirmed with a postoperative pathology report. None of the patients had a history of other types of malignant tumor, lymph node metastasis or distant metastasis, cardiovascular, thrombotic, liver failure or infection diseases. We also included 40 healthy individuals as the control group in our study. The individuals included in the control group were older than 18 years. In addition, these people had no diagnosis of acute infection, acute renal failure, liver failure, chronic disease or cancer throughout the study. During the follow-up of this study, the patients or their relatives were informed about the content of this study in detail and oral and written informed consent were provided. The study was approved by Ondokuz Mayıs University Ethics Committee. Pathological and clinical data of all patients were obtained completely from the hospital's medical records.

All blood test results, including serum fibrinogen and albumin levels, white blood cell count, and platelet count, were obtained within 2 weeks before surgery. Inflammatory indices were calculated using the following formulas: FAR = total fibrinogen/ total albumin; NLR = neutrophil count/lymphocyte count; LMR = lymphocyte count/monocyte count; PLR = platelet count/lymphocyte count; SII = platelet count × NLR; HRR = hemoglobin count/RDW count [21].

The patients were followed up every 3 months in the first 3 years and every 6 months thereafter. Patients were followed up with blood examination, biochemical tests, chest and abdominal CT(Computed Tomography). OS was calculated from the date of diagnosis to the date of death due to disease, and disease-free survival (DFS) was calculated from the date of diagnosis to the date of tumor recurrence.

### Statistical analysis

SPSS 24.0 software was used to analyze the data. The normal distribution of the data, arithmetic mean, ± standard deviation, and median minimum/maximum values were analyzed by Shappiro-wilk test. Student T test was used for the analysis of 2 groups with normal distribution, and Mann–Whitney **U** test was used for the analysis of 2 groups without normal distribution. Kaplan–Meier and Log-rank tests were used to evaluate survival. The best cut off value of the FAR was obtained using ROC curve analysis, and patients were divided into high- and low-FAR groups. The correlation between the preoperative FAR and clinicopathological features was analyzed with Log-rank test. *P* < 0.05 was accepted for statistical significance.

## Results

Of the 72 patients included in the study, 55 (%76.4) were male and 17 (%23.6) were female. Of the 40 individuals in our control group, 35 (%87.5) were male and 5 (%12.5) were female. The mean age in the patient group was 58.86 (min 34-max 82) and the mean age in the control group was 59.05 (min 32-max 81). Patients younger than 18 years of age were not included in the study. The patient group was selected from patients who had radical nephrectomy in the urology department between 2019 and 2021. Of the 72 patients, six died during the 24-month follow-up period. The average DFS was 34 months and the OS was 34 months as of the period of analysis. When the OS and DFS rates were compared with the Log Rank test, there was no significant difference between male and female patients (*p* > 0.05). All of the patients had undergone radical nephrectomy and their histological subtype was reported as clear cell carcinoma based on pathological findings. There were 61 (84.7%) patients with grade 1–2 and 11 (15.3%) patients with grade 3–4 according to Furhman Grade (FG). The distribution by tumor size was similar to the distribution set by FG. According to TNM staging, the number of patients with T1-2 was 61 (84.7%) and the number of patients with T3-4 was 11 (15.3%). Table 1 summarizes the detailed baseline characteristics of the selected patients.

**Table 1:** Comparison of clinicopathological data of different FAR groups

Features	Total	Preoperative FAR		*P* value
		≤ 0.114 (n = 53)	> 0.114 (n = 19)	
Age				0.938
≤ 65 years	50 (69.4)	34(73.9)	12 (26.1)	
> 65 years	22 (30.6)	19(73.1)	7(26.9)	
Gender				0.746
Male	55 (76.4)	41 (74.5)	14 (25.5)	
Female	17 (23.6)	12 (70.6)	5 (29.4)	
Fuhrman grade				< 0.0001
I–II	61 (84.7)	52(85.2)	9(14.8)	
III–IV	11 (15.3)	1 (9.1)	10 (90.9)	
Pathological T stage				< 0.0001
pT1–2	61 (84.7)	52 (85.2)	9(14.8)	
pT3–4	11 (15.3)	1(9.1)	10(90.9)	
Histology				
Clear cell carcinoma	72 (100.0)	53 (73.6)	19 (26.4)	
Non clear cell carcinoma	0 (0.0)	0 (0.0)	0 (0.0)	
Case survıval				< 0,0001
Exitus	6 ( 8.3)	0 (0.0)	6	
Alive	66 ( 91.7)	53	13	

Data are presented as n (%). FAR, fibrinogen-albumin ratio

Median score (min–max) NLR, PLR, HRR, LMR, SLL and FAR in all patient groups were 6.10 (1.17–25.37), 165.0650 (41.30–708.33), 0.9450 (0.44–1.38), 2.4100(0.42–32.87), 1,271,030(102,030–7,203,240) and 0.08000(0.007/0.286) respectively. In the control group, the Median score (min–max) of NLR, PLR, HRR, LMR, SLL and FAR were 1.4500 (0.34–4.69), 93.6250 (67.00–270.23), 1.0250 (0.57–1.35), 2.3000 (1.00–8.09), 505,975.00 (234,546–1,428,000) and 0.05450 (0.010–0.117) respectively (Table 2). NLR, PLR, HRR, SLL and FAR ratios between the patient and control groups were found to be significantly higher in the patient group when compared with the Mann–Whitney U test *p* < 0.001.

**Table 2:** Mean and median values of hematological parameters in patient and control groups

Group	Age	NLR	PLR	Hgb/RDW	LMR	SII	FAR
*Control*							
N	40	40	40	40	40	40	40
Mean	59.05	1.58	106.96	1.00	2.84	526,446.8	0.555
Std. Deviation	9.32	0.92	41.41	0.187	1.32	233,046.0	0.026
Median	60.0	1.45	93.62	1.02	2.30	505,975.0	0.054
Minimum	32	0.34	67.0	0.57	1.00	234,546	0.010
Maximum	81	4.69	270.23	1.35	8.09	1,428,000	0.117
*Patient*							
N	72	72	72	72	72	72	72
Mean	58.86	7.88	194.98	0.92	3.74	1,731,262	0.097
Std. Deviation	11.53	6.27	118.73	0.203	5.64	1,469,844	0.054
Median	59.0	6.1	165.07	0.94	2.41	1,271,030	0.08
Minimum	34	1.17	41.30	0.44	0.42	102,030	0.007
Maximum	82	25.37	708.33	1.38	32.87	7,203,240	0.286
*Total*							
N	112	112	112	112	112	112	112
Mean	58.93	5.63	163.54	0.94	3.42	1,300,971	0.082
Std. Deviation	10.75	5.88	106.84	0.20	4.59	1,318,051	0.0504
Median	59.0	2.94	130.32	0.97	2.34	676,058.5	0.071
Minimum	32	0.34	41.30	0.44	0.42	102,030	0.007
Maximum	82	25.37	708.33	1.38	32.87	7,203,240	0.286

The patients were divided into 2 groups as Grade 1–2 and Grade 3–4. When the NLR, PLR, HRR, LMR, SLL and FAR values of these two groups were compared with.

Mann–Whitney-U and Wilcoxon tests, PLR and FAR values were significantly higher in the Grade 3–4 group (*p* < 0.05). In addition, the patients were divided into two groups as T1-2 and T3-4. At the same time, PLR and FAR rates were significantly higher in T3-T4 patients (*p* < 0.05).

**Fig. 1:**
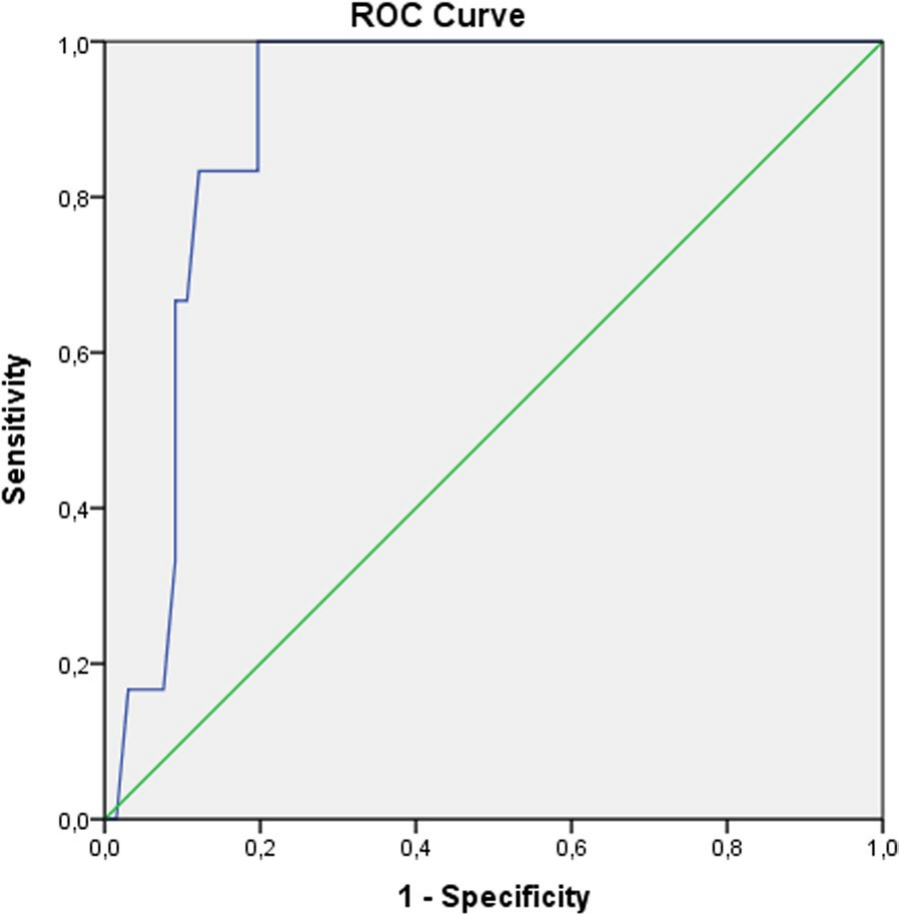
Determination of the cut-off value for the FAR by ROC curve analysis

We evaluated the effect of FAR on survival and death with Roc analysis. The best cutoff value for the preoperative FAR was determined by ROC curve analysis (Fig. 1). At a FAR of 0.114, sensitivity = 100%, specificity = 80% was found; therefore, 0.114 was selected as the cutoff value of the FAR (Area Under Curve (AUC): 0.900, Standart Error: 0.038, 95% CI 0.825–0.975, *P*:0.001).

Patients were divided into high and low FAR groups according to the cut-off value. The correlation between the preoperative FAR and clinicopathological features was analyzed by Log-rank test. A FAR > 0.114 was associated with higher Fuhrman grade (*P* < 0.0001) and later pathological T stage (*P* < 0.0001). Patients with a high FAR (> 0.114) had worse OS [ Std. Error 2.932, 95% CI 73.659–85.154, *P* < 0.0001]. In addition, in the present study, there was a significant relationship between the pathological grade of the patients and FAR, as well as a positive significant relationship between the platelet lymphocyte ratio and grade. (*p* < 0.020) Furthermore, a significant correlation was found between the pathology t stage of the patients and the platelet lymphocyte ratio. (*p*: 0,020).

## Discussion

FAR, which reflects the ratio of fibrinogen to albumin, has been a candidate to be a good prognostic factor in many cancers in recent years. In this regard, studies have been conducted on many cancers such as esophageal cancer [22], hepatocellular carcinoma [23], breast cancer [24], renal cell carcinoma [25], prostate cancer [26]. In this study, we aimed to investigate the prognostic significance of FAR in 72 patients after radical nephrectomy and also in our control group of 40 healthy individuals. Our study is a prospective study, and, to the best of our knowledge, there is no other study in literature with both a prospective and a control group, investigating the prognostic effect of FAR on RCC.

Nuclear grade, pathological stage, and pathological tumor type are commonly used indices to evaluate the prognosis of patients with RCC [27]. However, the effect of inflammation on tumor biology has been the subject of research for centuries. In general, inflammation is believed to affect every stage of tumor development, from tumor formation to metastases [28]. Pre- and post-operative inflammation indicators can predict the prognosis of the tumor, and these markers can guide the clinician in the treatment and follow-up of the disease.

Fibrinogen is not only a factor in coagulation, but also an acute phase reactive protein which is significantly increased in the case of systemic inflammation [15]. Systemic inflammation and coagulation are closely associated with tumor development [29]. Fibrinogen can be directly involved in cell angiogenesis, proliferation and distribution. It can do this by directly participating in the interaction between vascular endothelial growth factor, transforming growth factor-B, platelet-derived growth factor and fibroblast growth factor [30–32]. Albumıne which has been shown to decrease in inflamation is a negative acute phase reactant [16]. Inflammation and tumor formation and development reduce albumin levels, and as albumin decreases, the immune system may weaken and tumor development may accelerate [33]. We did not include patients with infectious disease, liver disease, known coagulation disorder and heart disease when choosing our patient group since albumin also decreases independently of the tumor in case of liver failure and infection.

In the present study, patients were divided into grades 1–2 and 3–4. In addition, according to the pathological tumor size, 2 separate groups were formed as T1-2 and T3-4. On the other hand, we also had a control group of 40 healthy individuals. When the NLR, PLR, HRR, SLL, FAR ratios between the patient group and the control group were compared with the Mann–Whitney U test, a significant increase was found in the patient group (*p* < 0.001). There was no additional disease in the healthy control group and the patient group and we found that FAR and other inflammatory blood indices were higher in the patient group. In different studies, these indices have been interpreted as useful prognostic factors in RCC [34, 35]. The number of patients with grade 3–4 in the patient group was 11 and the number of patients with grade 1–2 was 61. PLR and FAR rates were significantly higher in the grade 3–4 patient group (*p* < 0.05). Likewise, when the T1-2 and T3-4 groups were compared, the PLR and FAR ratios were found to be significantly higher in the T3-4 group (*p* < 0.05). It was assumed that the same results emerged due to the fact that patients with T3-4 and patients with grade 3–4 were in the same group.

In our patient group, we determined a cut-off value for FAR by ROC curve analysis (0.114). We divided the patients into 2 groups according to the FAR cut-off value. There was a significant correlation between the group with FAR > 0.114 and those with grade 3–4 and t3-4 (*p* < 0.0001), (*p* < 0.0001). OS was worse in patients with FAR > 0.114 (*p* < 0.0001).

In a study conducted by Jun Liu et al., similar results were obtained, and in this study, FAR and OS were compared among patients with grade 3–4 patients, but it was observed that FAR could not distinguish patients with worse OS. It was believed that this might result from the smaller number of grade 3–4 patient groups, as in our study. However, the prognostic effect of FAR was found to be more significant in the grade 1–2 subgroup. In other words, a patient with grade 1–2 with high FAR was believed to have worse OS, and it was recommended that these patients be followed more closely [25]. Since the number of patients was lower in our study, no significant relationship was found between FAR and OS in the subgroup analysis.

Ki-Tae Hwang et al. conducted a study on 793 patients with breast cancer and found that FAR was significantly higher in patients with stage 3–4, tumor size > 2 cm, and lymph node positive, and its prognostic importance was emphasized in these patients [24]. In this study, it was reported that the importance of FAR could not be determined clearly in subgroup analyses and further research was required on this subject.

There were some important limiting factors in the study of Jun Liu et al. The first of these was that the study was retrospective, which may have influenced patient selection results. Secondly, many factors such as acute and chronic infection and inflammation states and chronic liver diseases can affect FAR. These diseases and conditions were not excluded from the patient selection process. A third factor was that, as it was a retrospective study, many patients received different treatments for recurrence during follow-up, which may have affected OS. Although the number of patients was low in our study, acute and chronic infections, chronic and acute liver diseases, cardiovascular diseases and coagulation and thrombotic diseases which would affect FAR were excluded from the study. Our study is a prospective study and 6 patients died in the first 24 months during the 34-month follow-up, and the rest of the patients were followed throughout the study.

In our study, in addition to the FAR rate, the PLR rate was found to be significantly higher in both high grades and groups with high tumor size (*p*:0.020).

## Conclusions

Recently, the prognostic significance of FAR in various cancers has been investigated. In non-metastatic RCC patients, there is a need for indices which have prognostic importance to determine a cut-off during follow-up; to better categorize patients according to grade and tumor size; to reach early treatment and not to impair patients’ quality of life. Therefore, studies on FAR are very important. We believe that the present study will contribute to the studies conducted in order to determine the prognostic importance of FAR in RCC. In line with this study, perhaps cheaper and being more obtainable markers in the treatment renal cancer may come to fore. When the relationship between inflamation and cancer is further understood inflamation markers will be much more useful in the treatment of kidney cancer. However, due to the prospective nature of the present study, the number of cases is low, and studies with more patients are required for subgroup analysis.

## Data Availability

The datasets generated and/or analysed during the current study are not publicly available due [Since there are hospital data] but are available from the corresponding author on reasonable request.

## Abbreviations

RCC: Renal cell cancer
FAR: Fibrinogen/albumin ratio
ROC: Receiver operating characteristic
OS: Overall survival
FG: Fuhrman grade
CI: Confidence interval
NLR: Neutrophil–lymphocyte ratio
LMR: Lymphocyte-monocyte ratio
PLR: Platelet-lymphocyte ratio
SII: Systemic-immune-inflammation index
CRP: C-reactive protein
HRR: Hemoglobin-RDW (Red cell distribution width) ratio
CT: Computed tomography
DFS: Disease-free survival
TNM: Tumor, node, metastasis
AUC: Area under curve
